# Association between first trimester ambient temperature and the risk of gestational hypertension and pre-eclampsia: Insights from the PRECISE study cohort in The Gambia, Kenya and Mozambique

**DOI:** 10.21203/rs.3.rs-10471213/v1

**Published:** 2026-07-29

**Authors:** Cherlynn Dumbura, Tatenda Prestige Makanga, Stanley Luchters, Ana Bonell, Hawanatu Jah, Angela Koech, Anna Roca, Esperança Sevene, Moses Mukhanya, Herminio Cossa, Marie-Laure Volvert, Umberto D’Alessandro, Laura A Magee, Marleen Temmerman, Peter von Dadelszen

**Affiliations:** 1 Centre for Sexual Health and HIV AIDS Research (CeSHHAR) Zimbabwe; 2 Place Alert Labs, Midlands State University, Gweru, Zimbabwe; 3 Department of Public Health and Primary Care, Ghent University, Ghent, Belgium; 4 Liverpool School of Tropical Medicine (LSTM), United Kingdom; 5 London School of Hygiene and Tropical Medicine (LSHTM), Global Health Information Network; 6 Medical Research Council Unit, The Gambia at the London School of Hygiene and Tropical Medicine, Fajara, The Gambia; 7 Department of Obstetrics & Gynecology, Amsterdam University Medical Center, University of Amsterdam, Amsterdam Reproduction & Development Research Institute, Amsterdam, the Netherlands; 8 Centre of Excellence in Women and Child health, The Aga Khan University, Nairobi, Kenya; 9 ISGlobal, Hospital Clínic-Universitat de Barcelona, Barcelona, Spain; 10 Department of Physiological Sciences, Clinical Pharmacology, Faculdade de Medicina, Universidade Eduardo Mondlane, Maputo, Mozambique; 11 Centro de Investigacao em Saude de Manhica, Manhica, Mozambique; 12 Department of Women and Children’s Health, School of Life Course and Population Sciences, Faculty of Life Sciences and Medicine, King’s College London, London, United Kingdom; 13 International Centre for Reproductive Health, Ghent, Belgium; 14 ICREA, Barcelona, Spain;

## Abstract

**Background:**

Heat exposure during pregnancy has been linked to adverse maternal health outcomes. However, evidence regarding the relationship between ambient heat and hypertensive disorders of pregnancy (HDP) mixed with limited evidence from low-and middle-income countries (LMICs) in sub-Saharan Africa.

**Methods:**

We examined the association between ambient heat exposure and HDP among women in the PRECISE pregnancy cohort (The Gambia, Kenya and Mozambique). Logistic regression models estimated associations between pregnancy window and preconception pregnancy windows and risk of HDP.

**Results and Discussion:**

The cohort analysis subset HDP incidence was 15.9%. HDP status varies by maternal age, education, and other characteristics. Each 1°C increase in first-trimester temperature was associated with 8% increased odds of HDP (aOR 1.08, 95%CI:1.04 to 1.13, p<0.001). A similar relationship was observed for preconception heat exposure.

**Conclusion:**

First trimester heat exposure was associated with increased HDP risk, highlighting early pregnancy as a critical window for targeted interventions.

## One Sentence Summary

Every 1°C increase in first trimester heat exposure is associated with eight percent elevated risk of developing hypertensive disorders of pregnancy: gestational hypertension or preeclampsia in the PRECISE pregnancy cohort.

Global temperatures continue to rise, with populations worldwide experiencing more heatwave days compared to the period 1986 to 2005 and the year 2024 being stated as the hottest on record^1,2^. The continent of Africa in particular is experiencing some of the fastest increases in heat exposure^3^.

Extreme heat exposure has been linked to a range of adverse health outcomes including heat stroke, cardiovascular events, renal conditions and mortality^4–7^. The risk of adverse heat related health outcomes is higher for population groups whose physiological mechanisms to cool down may be compromised for example the elderly (reduced thermoregulatory reserve), children (body-size related heat gain and dependence on adults), pregnant women (increased metabolic and cardiovascular demand) and those with pre-existing comorbidities^8–10^.

Pregnant women are a particularly vulnerable population to heat due to major changes in their physiology during pregnancy, especially to placenta-related health outcomes such as preterm birth, stillbirths and hypertensive disorders of pregnancy (HDP). HDP, including chronic hypertension, gestational hypertension, and pre-eclampsia, remain leading causes of maternal morbidity and mortality^11^. Globally HDP accounts for almost 16 % of maternal deaths and contributes to severe maternal morbidity, preterm delivery, fetal growth restriction, and long-term cardiovascular risk for both mother and child^12,13^. Several biological mechanisms may link extreme heat exposure to hypertensive disorders of pregnancy (HDP). Heat exposure can induce maternal heat strain, adding to already increased cardiovascular demands of pregnancy. Heat-related dehydration and altered thermoregulation may also affect uteroplacental blood flow, particularly in women with hypertension or pre-eclampsia. In early pregnancy, heat-related cellular stress, systemic inflammation, oxidative stress and placental or endocrine disruption may interfere with placental development and vascular function, potentially increasing susceptibility to HDP. However, molecular pathways, including heat shock protein responses, remain poorly characterized in human pregnancy studies^14^.

Despite the growing body of climate-maternal health research, evidence regarding heat exposure and HDP remains mixed. Many prior studies have focused on late pregnancy or delivery period exposures, used coarse spatial exposure assignment or failed to account for residential mobility during pregnancy which is common custom in some African populations. Early pregnancy may be a particularly important exposure window as implantation and placentation occur during the first trimester^9,15–17^.

In this study we assessed the association between maximum ambient temperature exposure during preconception and the pregnancy trimester windows and the risk of gestational hypertension/pre-eclampsia in the PRECISE study, a multi-country African pregnancy cohort with detailed exposure assessment and longitudinal follow-up^18,19^.

## RESULTS

### Cohort characteristics

Among the 6,002 participants included in the univariate analysis, the median maternal age was 25 years [IQR 21 to 30] (N=5998), and 98.7% were of Black African descent. Marital status, religion and highest education attained differed significantly across study sites. Overall, 73.6% of the participants were housewives, while 26.4% reported being employed, including in informal employment.

Most participants were parous. In The Gambia 48.7% of women had more than three (3) previous births, compared with 19.7% in Mozambique and 25.3% in Kenya. Overall HIV prevalence in the analysis cohort was 5.5% with clear variation by country: 1.7% in The Gambia, 2.9% in Kenya and 11.3% in Mozambique. The median gestational age at delivery was 39.3 weeks [IQR: 37.7 to 40.7].

Gestational hypertension/pre-eclampsia status differed significantly by sociodemographic maternal characteristics: country, age, highest education level attained and residence (rural vs. urban or peri-urban) areas [p<0.05]. No statistically significant differences in HDP status were observed with respect to maternal dietary diversity, marital status, mother lives alone (Yes/No), tobacco product use at baseline (Table 1).

In terms of maternal health characteristics, gestational hypertension/pre-eclampsia status differed significantly by conception season [p<0.001], HIV status [p=0.004], number of fetuses in the index pregnancy [p= 0.05], maternal BMI [p<0.001], average maternal mid upper arm circumference (MUAC) [p<0.001], and history of pregestational diabetes (Table 2).

No significant differences between women with gestational hypertension/pre-eclampsia and normotensive women were observed for parity and gestational age at delivery [p>0.05] (Table 2).

### First-trimester ambient temperature and hypertensive disorders of pregnancy [DAG and apriori adjusted model]

In the first trimester exposure adjusted logistic regression model, each 1°C increase in average maximum ambient temperature a woman had 8% increased odds of developing gestational hypertension/pre-eclampsia adjusted Odds Ratio (aOR) (95%CI):1.08 (1.04 – 1.13) [p < 0.001] (Table 3).

### Sensitivity analyses

#### First-trimester ambient temperature and hypertensive disorders of pregnancy [DAG and apriori adjusted model and climate zones]

In the first trimester exposure adjusted logistic regression model adjusted for climate zones, each 1°C increase in average maximum ambient temperature a woman had 8% increased odds of developing gestational hypertension/pre-eclampsia adjusted Odds Ratio (aOR) (95%CI):1.08 (1.04 –1.13) [p < 0.001].

#### Sub-analysis by country: First-trimester ambient temperature and hypertensive disorders of pregnancy [DAG and apriori adjusted model]

Country-stratified analyses showed increased odds of developing gestational hypertension/pre-eclampsia per 1°C increase in trimester one average temperatures, further supporting the robustness of the findings, Kenya aOR (95%CI): 1.08 (1.01–1.15) [p= 0.02], Mozambique aOR 1.08 (95%CI: 1.01–1.16)[p=0.02], and The Gambia aOR (95%CI): 1.07 (1.00–1.15) [p=0.09]. Although confidence intervals widened in site-specific models due to reduced sample size, effect estimates were broadly consistent across countries (Table 4).

### DAG covariate adjusted model: First-trimester ambient temperature and hypertensive disorders of pregnancy

The model adjusted only for the DAG-specified variables (country, conception season (25th percentile vs 75th percentile temperatures), Gramin poverty index) produced results similar to the fully adjusted model for each 1°C increase in trimester one average temperature a woman had 8% increased odds of developing gestational hypertension/pre-eclampsia aOR (95%CI):1.08 (1.04 –1.12) [p<0.001]. This similarity in the findings suggests that the heat and gestational hypertension/pre-eclampsia estimated association was robust to the inclusion of additional predictors (Supplementary Table 1).

### Sub-analysis by gestational age dating method

Overall, the subgroup findings suggested that the direction of association between first trimester heat exposure and hypertensive disorders of pregnancy differs by gestation age dating methods. Where ultrasound-based dating was available, the estimates provided the strongest basis for interpretation because ultrasound is generally considered a more reliable method for gestational age N=1967, aOR 1.12(95%CI: 1.06;1.88) [p<0.001]. Interestingly symphysis-fundal height (SFH) subgroup also had increased risk and was statistically significant despite the small sample size N=461, aOR 1.16(95%CI: 1.02;1.32) [p=0.020] (Supplementary Table 1).

### Preconception, Second-trimester two and Third-trimester three ambient temperature exposure and HDP analysis

In the analysis examining association between ambient temperature exposure experienced during the preconception period, second trimester and third trimester and odds of developing hypertensive disorders of pregnancy, unadjusted models showed with an elevated risk of gestational hypertension/pre-eclampsia per 1°C rise in temperature.

The preconception period exposure association remained positively associated with increased odds of developing gestational hypertension/pre-eclampsia aOR 1.05 (95%CI:1.01 – 1.08 [p=0.02]), whereas associations for second trimester exposure; and for the third trimester

After adjustment for DAG-specified and a priori variables, the associations for the second and third trimesters were attenuated and no longer statistically significant, aOR 1.01 (95%CI:0.97–1.05, [p= 0.68]) and aOR 0.96 (95%CI:0.93–1.00, [p=0.05]) respectively. (Supplementary Table 2).

## DISCUSSION

Our study indicates that ambient heat exposure in early pregnancy (i.e., first trimester) was associated with increased odds of gestational hypertension and preeclampsia. Specifically, the odds of developing hypertensive disorders increased by 8%, for each 1°C increase after adjusting for sociodemographic and health variables. This finding supports early pregnancy as a potentially sensitive window in which exposures may influence later HDP risk. Although the effect size appears modest at the individual level, a population-wide shift in heat exposure could translate into a meaningful increase in HDP burden, especially in settings with high baseline maternal risk. The biological interpretation is plausible, although not directly tested in this study. Early pregnancy is marked by trophoblast invasion, placental vascular development and maternal cardiovascular adaptation; heat-related oxidative stress, inflammatory signaling and heat-induced endothelial dysfunction may disrupt these processes^20,21^. Inflammatory signaling such as activation of maternal early trimester heat shock proteins after heat exposure has also been found to occur in women who later developed pre-eclampsia at 34 weeks gestation^22^.

Our findings are broadly consistent with a growing body of evidence linking higher ambient temperature during early pregnancy with HDP risk. Lakhoo et al., 2024 reported that hypertensive disorders of pregnancy were linked with heat exposure in 21 of 28 studies and Mao et al.,2023 found that heat exposure in the first half of pregnancy increased risk of pre-eclampsia/eclampsia with pooled OR of around 1.54 (95% CI:1.10, 2.15)^13,23^.

The direction of the association is consistent with Shashar et al.,2020 study carried out in Southern Israel from a retrospective cohort, who reported a 36% increase in pre-eclampsia risk per overall elevation of one IQR (~9°C) increase in first-trimester temperature RR 1.36 (95%CI:1.15–1.61) with increasing first-trimester temperature.

A Johannesburg time-to event study carried out in a South African urban setting found the third and fourth gestational week as high-risk windows for maternal hypertensive disorders aHR:1.79 (95%CI: 1.19–1.21), and findings from a Chinese national cohort found that ambient temperature was linked to eclampsia/preeclampsia and gestational hypertension, with heat exposure in the first half of pregnancy increasing odds (aOR1.16 (95%CI: 1.10–1.22)^16,21^.

Our findings are consistent with the clinical review by (Magee et al., 2022) which highlights early pregnancy as an important window of risk identification and prevention. This includes the timely initiation of low-dose aspirin among women with high-risk of pre-eclampsia, particularly given that early-onset pre-eclampsia is often linked to impaired placentation and uteroplacental dysfunction^24^.

Therefore, the first trimester is emerging as a crucial exposure window of interest that needs to be strongly considered for policy development. These findings suggest that heat exposure could be considered in antenatal risk assessment, counselling and heat-health messaging particularly during early pregnancy.

However, the literature is not fully consistent, there are differences across studies which may reflect variation in climate context, temperature range, exposure metrics, outcome definitions, gestational windows and statistical modelling approaches.

Our results contrast with studies by Neela and Ramman, 1993 (India) and Immink et al.2008, which reported either null or protective associations between heat and gestational hypertension. These differences may arise due to variations in study context, exposure assessment (seasonal influence instead of ambient temperature), and the outcome being pre-eclampsia admissions analytical methods. For example, Neela found no association between season and pre-eclampsia admissions. This null finding may reflect the limited temperature variation (1.6°C) between monsoon and dry seasons as well as the study’s small sample size^25,26^.

A key strength of this study is its multi-country, multi-site design, which captures variations across urban, peri-urban and rural settings. enhances the generalizability of our findings by capturing data from diverse environmental and healthcare settings including both urban, peri-urban and rural contexts in the areas under investigation in this study. The use of standardized sociodemographic and health data collection across the three countries allowed for analytical adjustment for important individual level risk factors.

In addition, the study uses individually assigned village-level exposure data, reducing exposure misclassification compared with studies that assign temperature exposure at the city or health facility level. The exposure assignment used in this study allows an understanding of how heat influences maternal health outcomes layering potential migration during the follow-up period of participants.

Some limitations should be considered when interpreting these findings. The recruitment took place in specific regions, which may limit the generalizability of our findings to other settings in sub-Saharan Africa with different climate zones, sociodemographic and health services. Data collection was paused during the COVID-19 pandemic, which interrupted the continuity of the study this may have affected follow-up completeness, visit-timing and opportunity to detect HDP outcomes consistently. We did not adjust for humidity or composite heat stress indices heat index or wet-bulb globe temperature, which may better capture physiological heat strain than ambient temperature alone but allowed for comparison of findings with previous studies in this research area. Because diagnosis timing was based on the visit at which HDP was first recorded, the actual onset of disease may have occurred before the recorded visit. As a result, the precise timing of the HDP onset could not be established; it could only be approximated within the gestational age window of the visit at which HDP was first recorded. We therefore analysed hypertensive disorder as a binary health outcome, rather than applying a time to event approach. Logistic regression was used because available data supported outcome classification but not reliable estimation of disease onset. Findings should therefore be interpreted as associations with HDP occurrence, rather than precise estimates of time to HDP onset.

Overall, this study adds to emerging evidence that early pregnancy may be a sensitive window for heat-related HDP risk. The findings are biologically plausible, consistent with several studies showing higher HDP risk after early pregnancy heat exposure, and relevant for climate sensitive antenatal care in sub-Sharan African settings. Future work should assess gestational timing in more detail, compare heat metrics, and examine whether humidity, baseline maternal risk and access to antenatal care can modify heat-related HDP risk.

In conclusion, our study provides evidence that increased heat exposure during the first trimester is associated with higher odds of hypertensive disorders of pregnancy in the PRECISE cohort. The findings add to the growing evidence on heat-related maternal health risks and highlight early pregnancy as an important period for prevention, screening and intervention in sub-Saharan Africa.

The results have implications on policy, clinical practice, health system preparedness, and community-level interventions in sub-Saharan Africa. Public health strategies aimed at reducing the burden of hypertensive disorders of pregnancy should prioritise early antenatal care, particularly during the first trimester to ensure early identification of women at higher risk.

Our findings suggest that public health policies focused on improving gestational hypertensive outcomes should prioritize early pregnancy, particularly the first trimester. This has significant implications for maternal health, especially in resource-limited settings where the additional burden to specialized obstetric care could arise.

## METHODS

### Study design and climatic setting

The PREgnancy Care Integrating Translational Science Everywhere (PRECISE) study was a prospective observational pregnancy cohort investigating three placental pregnancy complications: hypertension, fetal growth restriction and stillbirth. The study was conducted across three countries The Gambia, Kenya, and Mozambique, each country representing different geographic and climatic environments. Recruitment sites included from The Gambia: Farafenni District hospital(urban) and two rural primary healthcare facilities in Illiasa and Nyagen Sanjal. In Kenya, recruitments occurred at Mariakani Sub-County Hospital (urban) and Rabai Sub-County Hospital (rural), both in Kilifi County, coastal Kenya. In Mozambique, recruitment occurred at Manhiça District Hospital (urban) and Xinavane Rural Hospital (rural) in Maputo Province^18^.

In terms of climate, The Gambia’s Northbank region has a wet season from June to December and a long dry season starting in January. Average temperatures are around 30°C during the dry season and 27°C in the wet season.^27^.

Kenya’s Kilifi County has mean annual temperatures ranging from 26°C to 30 °C and monthly average highs of 28°C to 30°C^7^. Rainfall patterns are influenced by Indian ocean monsoons with a tropical savanna climate starting in March (spring) followed by a dry season from May to September^28^.

Maputo Province (Manhica and Xinavane) in Mozambique has a tropical climate with a hot-wet season from October to March and a cool-dry season from April to September^22^. Average annual temperatures range from 25°C to 30°C with the hotter months (summer) at the higher end and the cooler dry season (winter) at the lower end^30^.

Recruitment for the PRECISE study occurred between January 2019 and December 2022, enrolling a total of 6770 pregnant women who had come for their first antenatal care (ANC) booking and were planning to deliver at the respective study health facilities. The study included four scheduled visits: two antenatal visits (the first during the ANC booking and the second in the third trimester, at least 8 weeks later), a visit during delivery, and a final visit between 6 weeks and 6 months postpartum^18^.

Clinicians present at each follow-up visit measured blood pressure readings and hypertensive diagnoses statuses were then classified according to standard definitions for chronic hypertension, gestational hypertension, and pre-eclampsia^24–29^.

### Ethics

Research ethics approval for the study was obtained from This study involves human participants. Approval for the PRECISE study was obtained in King’s College London (Ref HR-17/18–7855), Aga Khan University Hospital (Ref 2018/REC-74), The Gambia Government/MRC Joint Ethics Committee (Ref SCC 1619) and the Mozambique Ministry of Health, National Bioethics Committee for Health (545/CNBS/18). Participants gave written informed consent to participate in the study before taking part.

### Outcomes

A Microlife CRADLE Vital Signs Alert semi-automated device was used for clinical blood pressure measurements at all visits^31^. The primary outcome was a composite indicator of gestational hypertension or pre-eclampsia arising during pregnancy. Gestational hypertension was defined as new-onset hypertension (systolic ≥140 mmHg and/or diastolic ≥90 mmHg) at least 20+0 weeks’ gestation. Pre-eclampsia was defined as gestational hypertension, and significant proteinuria ≥2+) or evidence of end-organ dysfunction (abnormal symptoms [e.g., headache, visual disturbance, right upper quadrant pain], signs [e.g., eclampsia], laboratory tests [e.g., thrombocytopenia, transaminitis], or evidence of uteroplacental dysfunction [e.g., stillbirth, small-for-gestational age infant])^24^. Women with chronic hypertension at enrolment, defined as hypertension present before pregnancy, diagnosed before 20 weeks of gestation or persisting beyond 12 weeks postpartum, were excluded from the analysis. Date of conception was estimated using the date of delivery and gestational age at delivery. Gestational age was determined based on the reported date of last menstrual period and/or ultrasound assessment conducted during early or late pregnancy. For some participants gestational age was determined by the TraCer (transcerebellar diameter) app that dates pregnancies using the highly conserved and growth restriction resistant transcerebellar diameter^32^.

### Heat exposure assessment

Women’s residence was recorded during each visit, with villages classified as urban, peri-urban, or rural using the Global Human Settlement Layer. Residential location was updated at each follow-up visit to account for migration, which is common in many African settings during pregnancy.

Daily maximum ambient temperature was obtained from the ERA5 reanalysis dataset through Google Earth engine. We extracted daily maximum 2m air temperature at a 0.25° × 0.25° spatial resolution (~31km) using participants’ residential village polygons. Temperatures were converted from Kelvin to degrees Celsius before linking them to participant data using the woman’s exposure period (pregnancy duration). The gestational period for each woman was broken down into respective trimesters and preconception period. Trimester and preconception average maximum ambient temperatures for each participant were derived and assigned.

### Data

**Fig. 1:**
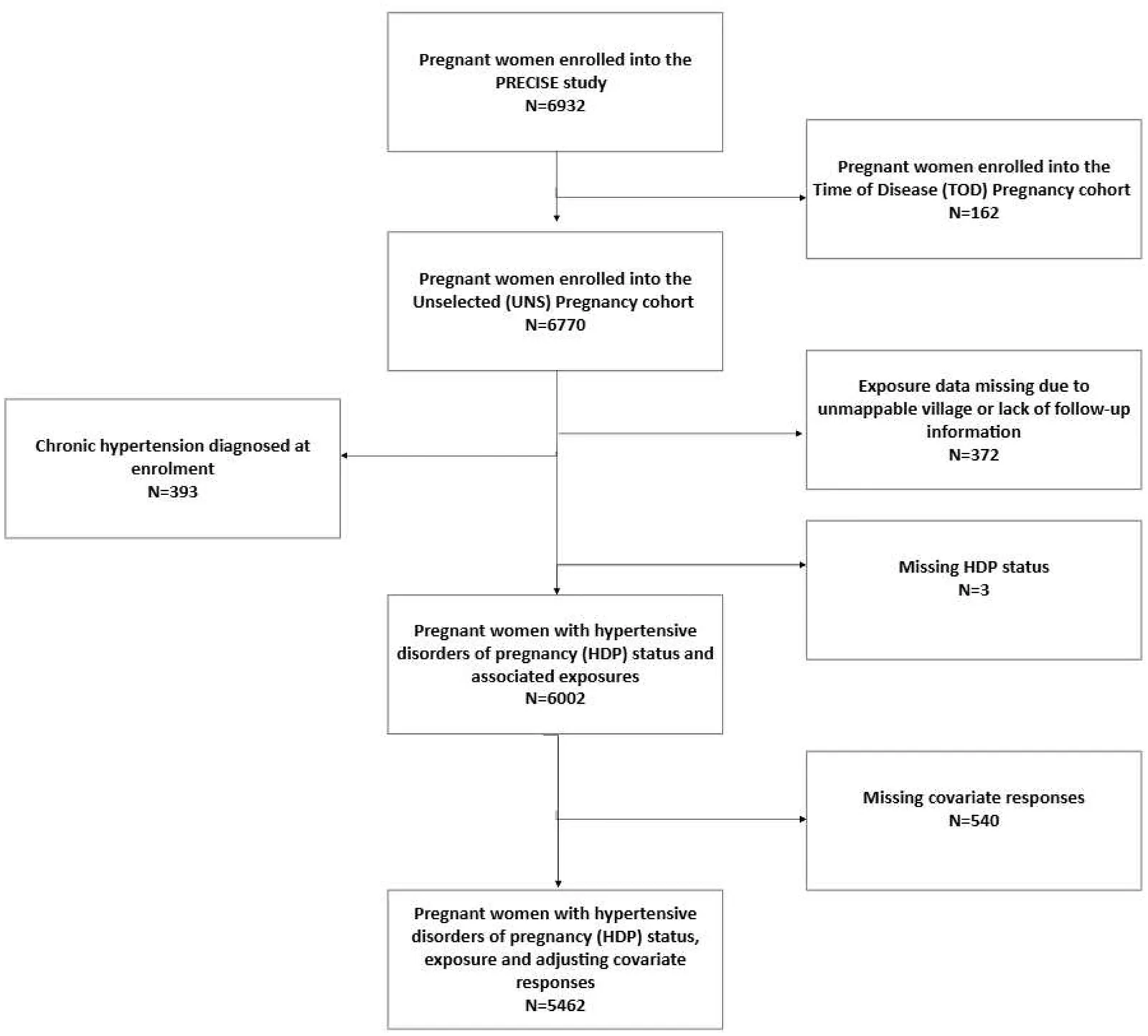
Study cohort UNS from enrolment to delivery visit Updating of numbers(N) in Figures and Tables progress

From the 6770 pregnant women enrolled, 372 women were excluded for missing exposures or missing follow-up information (dates or vague locations instead of specific village names, 393 women were excluded for a positive chronic hypertension diagnosis at enrolment, and 3 were excluded for missing gestational hypertension/pre-eclampsia status information. 6002 women were included in the unadjusted analysis and after dropping missing sociodemographic and health covariates, 5462 women were included in the adjusted analysis (Fig. 1).

### Statistical Analysis

**Fig. 2:**
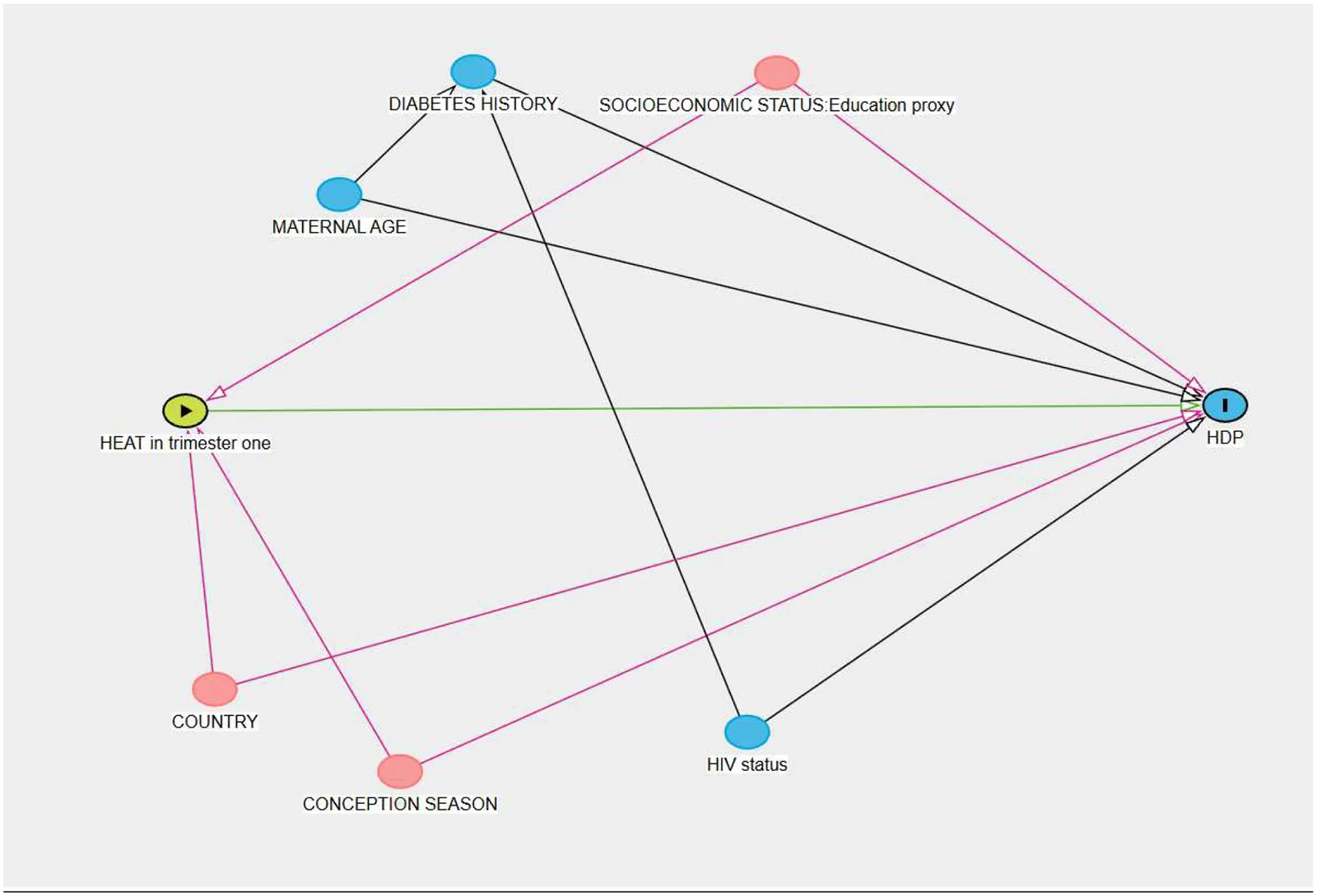
Direct Acyclic graph with minimal adjusting set stated as: country, conception season, socioeconomic status (Gramin index)

Cohort characteristics were summarised by gestational hypertension/pre-eclampsia status using Chi-square or Fisher’s exact test for categorical variables based on the minimum responses per category and the student’s t-test or Wilcoxon rank test depending on skewness of continuous variable distributions. Covariate selection was guided by a directed acyclic graph (DAG), Fig. 2, which identified the minimal sufficient adjustment set for estimating total effect of heat on gestational hypertension/pre-eclampsia. Minimal adjustment set covariates identified were conception season, country and socio-economic status (Grameen poverty index). A priori variables that have previously been reported as confounders of the heat-HDP association in previous studies and/or were significant in the descriptives of this study (maternal age, maternal tobacco product use, average MUAC, whether mother lives alone, parity and HIV status) were also included in the adjusted analyses.

Logistic regression was used to estimate the association between average maximum first-trimester temperature and gestational hypertension/pre-eclampsia adjusted for country (The Gambia/Kenya/Mozambique), conception season(hot/moderate/cold), and socioeconomic status(Grameen poverty index score), HIV status(Positive/negative), maternal age at enrolment, average maternal MUAC, maternal reported tobacco product use(yes/no), whether mother lives alone(yes/no) and parity (nulliparous, parity level 1, 2 and >=3).

### Sensitivity analysis

#### Sub-analysis by country

Country stratified analyses of the primary logistic regression model were carried out to assess consistency across settings.

#### DAG covariate adjusted model

A model adjusting for the DAG selected variables was run to assess robustness and a model that included climate zones to assess if the findings changed after accounting for the different climatic zones of the study sites.

#### Sub-analysis by gestational age dating method

Sub-analysis if the primary logistic model was applied to subgroups of the population according to the method used for gestational age dating: Ultrasound, Last menstrual period (LMP), symphysis-fundal height (SFH), Tracer and missing/outlier gestational age records. This analysis was used to assess whether the association between heat exposure and hypertensive disorders was consistent across gestational age during dating methods, and to examine the potential influence of gestational age classification on the main findings.

## Supplementary Material

1

## Figures and Tables

**Table 1:** Sociodemographic characteristics PRECISE pregnancy cohort study participants with exposures available at enrolment by either gestational hypertension or pre-eclampsia during study follow-up period) [chronic hypertension population has been excluded] N=6,002

Population characteristics	Normotensive pregnant women [N=5046] N (%) / median (IQR) / median (IQR)	[GH or PE] [N=956] N (%) / median (IQR) / mean (SD)	*P*-value
**Maternal age, mean years (SD) N=5998**	25 (21;30)	**26 (21;31)**	0.008
**Maternal Country of residence**			
• Kenya	2528 (50.1)	435 (45.5)	<0.001
• Mozambique	1723 (34.2)	279 (29.2)	
• The Gambia	795 (15.8)	242 (25.3)	
**Mother's highest school level attained** *N=6002			<0.001
• Tertiary	348 (6.9)	72 (7.5)	
• Secondary	1805 (41.2)	335 (35.0)	
• Primary	2077 (35.8)	335 (35.0)	
• None	815 (16.2)	214 (22.38)	
32/4999Mother's religion *N=5999	3210 (63.7)	541 (56.6)	<0.001
• Christian	1800 (35.7)	413 (43.2)	
• Muslim	33 (0.7)	2 (0.2)	
• Other			
**Maternal rural-urban index, N=6002**			
• Rural	1028 (20.4)	215 (22.5)	<0.010
• Peri-urban	1485 (29.4)	248 (25.9)	
• Urban	2533 (50.2)	493 (51.6)	
**Maternal marital status** *N=5991			
• Married/Cohabitating	4078 (81.0)	792 (82.9)	0.400
• Single/Never married	918 (18.2)	157 (16.4)	
• Separated/Divorced	39 (0.8)	7 (0.7)	
**Primary construction material of mother's house (exterior walls)** *N=5997			
• Brick	3318 (65.8)	659 (69.0	0.044
• Stone	903 (17.9)	171 (17.9)	
• Earth and mud	540 (10.7)	74 (7.7)	
• Other	280 (5.6)	52 15.4)	
Mother meets minimum Dietary Diversity score *N=5609			
• Yes	2338 (52.4)	449 (50.11)	0.216
• No	2126 (47.6)	447 (49.9)	
**Mother lives alone, N=5988**			
• **Yes**	229 (4.6)	55 (5.8)	0.110
• **No**	4805 (95.5)	899 (94.2)	
Maternal current use of tobacco products *N=5983			
• Yes	32 (0.60)	11 (1.2)	0.082
• No	4999 (99.4)	941 (98.8)	

Number(N) and column % of total, or summary statistics specified. Abbreviations: GH: Gestational hypertensive disorder, E: Eclampsia, PE: Pre-eclampsia, HIV: Human Immuno-deficiency virus, IQR: interquartile range CI confidence interval. a Chi-squared test, b t- test, c Mann-Whitney U test,

**Table 2:** Health and pregnancy characteristics of PRECISE pregnancy cohort study participants with exposures available at enrolment by either gestational hypertension or pre-eclampsia during study follow-up period) [chronic hypertension population has been excluded], N= 6,002

Population characteristics	Normotensive pregnant women (N=5046) N (%) / median (IQR) / mean (SD)	GH or PE (N=956) N (%) / median (IQR) / mean (SD)	P-trend
**Maternal HIV status, N=6002**			
• Negative	4749 (94.1)	922 (96.4)	0.004
• Positive	297 (5.9)	34 (3.6)	
Maternal BMI *N=5994			
• <18.5	254 (5.0)	45 (4.7)	<0.001
• 18.5–24.9	2990 (59.4)	463 (48.4)	
• 25.0–29.9	1306 (25.9)	274 (28.7)	
• 30+	488 (9.7)	174 (18.2)	
**Maternal BMI median (IQR)**	23.6 (21.2–26.4)	24.6 (21.6–28.5)	<0.001
Maternal mid upper arm circumference *N=5995 median (IQR)	26.6 (24.4;29.1)	27.2 (25.2;30.6)	<0.001
**Pregestational diabetes, N=6002**			
• Yes	21 (0.4)	9 (0.94)	0.035
• No	5025 (99.6)	947 (99.1)	
Gestational age at delivery: *N=5114 median (IQR)	39.3 (37.7–40.6)	39.3 (37.7–40.7)	0.340
**Conception season (25**^**th**^ **percentile vs 75**^**th**^ percentile) *N=5491			
• Cold	1449 (31.4)	220 (25.1)	<0.001
• Moderate	2225 (48.2)	442 (50.5)	
• Hot	942 (20.4)	213 (24.3)	
Number of fetuses index pregnancy *N=5159			
• Singleton	4164 (98.7)	921 (97.9)	0.05
• Multiple	54 (1.3)	20 (2.1)	
**Parity, N=6002**			
• 0	1570 (31.1)	320 (33.5)	0.318
• 1	1244 (24.7)	213 (22.3)	
• 2	851 (16.9)	156 (16.3)	
• >=3	1381 (27.4)	267 (27.9)	

Number(N) and column % of total, or summary statistics specified. Abbreviations: GH: Gestational hypertensive disorder, E: Eclampsia, PE: Pre-eclampsia, HIV: Human Immuno-deficiency virus, IQR: interquartile range, CI confidence interval. a Chi-squared test, b t- test, c Mann-Whitney U test,

**Table 3:** Table showing unadjusted and adjusted risk of gestational hypertension and pre-eclampsia per degree increase in the average maximum ambient temperature experienced in trimester one for the PRECISE pregnancy cohort, unadjusted analysis N=6002, adjusted analysis N=5462

Model	Measure of Association (95% CI)	P-value
Unadjusted analysis	OR (95%CI)	P-Value
Odds of gestational hypertension or pre-eclampsia disorders degree increase in mean temperature in trimester one, (N = 6002)	1.11 (1.09 – 1.14)	<0.001
Multivariate analysis:	aOR ^c^ (95% CI)	P-value
Odds of gestational hypertension or pre-eclampsia per degree increase in mean temperature in trimester one Adjusted for DAG and a priori variables: (Model adjusted for country, maternal age, Gramin poverty index, conception season (25^th^ vs 75^th^), mother lives alone(yes/no), parity, maternal MUAC and HIV status N = 5462	1.08 (1.04 – 1.13)	<0.001

Number (N) observations remaining in model, Measure of association **Odds Ratios** per model and respective 95% confidence intervals, p-values. Adjusted model covariates are indicated in each model.

**Table 4:** Table showing adjusted risk of gestational hypertension or pre-eclampsia per degree increase in the average maximum ambient temperature experienced in trimester one by country for the PRECISE pregnancy cohort, N = 5471

Odds of hypertensive disorders per degree increase in average maximum ambient temperature in trimester one stratified by country		aOR (95% CI)	P-value
Kenya	N=2595	1.08 (1.01, 1.15)	0.020
Mozambique	N=1848	1.08 (1.01; 1.16)	0.019
The Gambia	N=1018	1.07 (0.99; 1.15)	0.092
(adjusted for country, conception season (cold/ moderate/ hot) Gramin poverty index, maternal HIV status, maternal, maternal average MUAC, maternal reported use of tobacco use product, mother lives alone and parity categorised)			

Number (N) observations remaining in model, Measure of association **Odds Ratios** per model and respective 95% confidence intervals, p-values. Adjusted model covariates are indicated in each model.

## Data Availability

The data generated in this study are available upon request and Data Access committee approval. The code is available on request.

## References

[1] WHO. Climate change and Health. WHO https://www.who.int/news-room/fact-sheets/detail/climate-change-and-health (2023).

[2] NASA. Temperatures Rising: NASA Confirms 2024 warmest year on record. NASA https://www.nasa.gov/news-release/temperatures-rising-nasa-confirms-2024-warmest-year-on-record/ (2025).

[3] Report I. S. Summary for Policymakers of the Intergovernmental Panel on Climate Change. 402, 2346–2394 (2024).

[4] Basu R. & Samet J. M. Relation between elevated ambient temperature and mortality: A review of the epidemiologic evidence. Epidemiol. Rev. 24, 190–202 (2002). 12762092 10.1093/epirev/mxf007

[5] Gasparrini A. Mortality risk attributable to high and low ambient temperature: A multicountry observational study. Lancet 386, 369–375 (2015). 26003380 10.1016/S0140-6736(14)62114-0 PMC4521077

[6] de Bont J. Impact of heatwaves on all-cause mortality in India: A comprehensive multi-city study. Environ. Int. 184, 108461 (2024). 38340402 10.1016/j.envint.2024.108461 PMC11790314

[7] Scorgie F. “Mothers get really exhausted!” The lived experience of pregnancy in extreme heat: Qualitative findings from Kilifi, Kenya. Soc. Sci. Med. 335, 116223 (2023). 37725839 10.1016/j.socscimed.2023.116223

[8] Oudin Åström D., Bertil F. & Joacim R. Heat wave impact on morbidity and mortality in the elderly population: A review of recent studies. Maturitas 69, 99–105 (2011). 21477954 10.1016/j.maturitas.2011.03.008

[9] Chersich M. F. Increasing global temperatures threaten gains in maternal and newborn health in Africa: A review of impacts and an adaptation framework. Int. J. Gynecol. Obstet. 160, 421–429 (2023). 10.1002/ijgo.14381 PMC10087975 35906840

[10] Brink N. Impacts of heat exposure in utero on long-term health and social outcomes: a systematic review. BMC Pregnancy Childbirth 24, 1–24 (2024). 38704541 10.1186/s12884-024-06512-0 PMC11069224

[11] Magee L. A., Pels A., Helewa M., Rey E. & Von Dadelszen P. Diagnosis, evaluation, and management of the hypertensive disorders of pregnancy. Pregnancy Hypertens. 4, 105–145 (2014). 26104418 10.1016/j.preghy.2014.01.003

[12] Magee L. A. Can adverse maternal and perinatal outcomes be predicted when blood pressure becomes elevated? Secondary analyses from the CHIPS (Control of Hypertension In Pregnancy Study) randomized controlled trial. Acta Obstet. Gynecol. Scand. 95, 763–776 (2016). 26915709 10.1111/aogs.12877 PMC5021204

[13] Lakhoo D. P. A systematic review and meta-analysis of heat exposure impacts on maternal, fetal and neonatal health. Nat. Med. 31, 684–694 (2025). 39500369 10.1038/s41591-024-03395-8 PMC11835737

[14] Bonell A. Physiological and biological response to heat exposure in pregnancy: a systematic review and meta-analysis. Lancet Planet. Heal. 10, 1–15 (2026). 10.1016/j.lanplh.2026.101431 42127955

[15] Melo B., Amorim M., Katz L., Coutinho I. & Figueiroa J. N. Hypertension, pregnancy and weather: Is seasonality involved? Rev. Assoc. Med. Bras. 60, 105–110 (2014). 24918996 10.1590/1806-9282.60.02.006

[16] Part C. Ambient temperature during pregnancy and risk of maternal hypertensive disorders: A time-to-event study in Johannesburg, South Africa. Environ. Res. 212, 15–17 (2022). 10.1016/j.envres.2022.113596 35661733

[17] Shashar S. Temperature and preeclampsia: Epidemiological evidence that perturbation in maternal heat homeostasis affects pregnancy outcome. PLoS One 15, 1–14 (2020). 10.1371/journal.pone.0232877 PMC7234374 32421729

[18] Craik R. PREgnancy Care Integrating translational Science, Everywhere (PRECISE): a prospective cohort study of African pregnant and non-pregnant women to investigate placental disorders – cohort profile. BMJ Open 15, 1–13 (2025). 10.1136/bmjopen-2024-091831 PMC12067852 40350193

[19] Craik R. PRECISE pregnancy cohort: Challenges and strategies in setting up a biorepository in sub-Saharan Africa. Reprod. Health 17, 1–9 (2020). 32354368 10.1186/s12978-020-0874-7 PMC7191687

[20] McElwain C. J., Tuboly E., McCarthy F. P. & McCarthy C. M. Mechanisms of Endothelial Dysfunction in Pre-eclampsia and Gestational Diabetes Mellitus: Windows Into Future Cardiometabolic Health? Front. Endocrinol. (Lausanne). 11, 1–19 (2020). 33042016 10.3389/fendo.2020.00655 PMC7516342

[21] Xiong. Association between ambient temperature and hypertensive disorders in pregnancy in China. 1–11 (2020) doi:10.1038/s41467-020-16775-8. PMC7286884 32522990

[22] Robellada-Zárate C. M. First-trimester plasma extracellular heat shock proteins levels and risk of preeclampsia. J. Cell. Mol. Med. 27, 1206–1213 (2023). 37002651 10.1111/jcmm.17674 PMC10148059

[23] Mao Y. Associations between extreme temperature exposure and hypertensive disorders in pregnancy: a systematic review and meta-analysis. Hypertens. Pregnancy 42, (2023). 10.1080/10641955.2023.2288586 38053322

[24] Magee L. A. Preeclampsia. Journalism 11, 369–373 (2022).

[25] Neela J., R. l. Seasonal trends in occurrence of eclampsia. Natl. Med. J. India 34, 229–235 (1993). 8453355

[26] Immink A., Scherjon S., Wolterbeek R. & Steyn D. W. Seasonal influence on the admittance of pre-eclampsia patients in Tygerberg hospital. Acta Obstet. Gynecol. Scand. 87, 36–42 (2008). 17963049 10.1080/00016340701743066

[27] Sonko E., Tsado D. N., Yaffa S., Okhimamhe A. A. & Eichie J. Wet and Dry Season Effects on Select Soil Nutrient Contents of Upland Farms in North Bank Region of the Gambia. Open J. Soil Sci. 06, 45–51 (2016).

[28] Camberlin P. Climate of Eastern Africa To Cite This Version : HAL Id : Hal-04314379. Oxford Research Encyclopedia of Climate Science, (2023).

[29] von Dadelszen P. The Community-Level Interventions for Pre-eclampsia (CLIP) cluster randomised trials in Mozambique, Pakistan, and India: an individual participant-level meta-analysis. Lancet 396, 553–563 (2020). 32828187 10.1016/S0140-6736(20)31128-4 PMC7445426

[30] Mavume A. F., Banze B. E., Macie O. A. & Queface A. J. Analysis of climate change projections for mozambique under the representative concentration pathways. Atmosphere (Basel). 12, (2021).

[31] Von Dadelszen P. The PRECISE (PREgnancy Care Integrating translational Science, Everywhere) Network’s first protocol: Deep phenotyping in three sub-Saharan African countries. Reprod. Health 17, 1–15 (2020). 32354357 10.1186/s12978-020-0872-9 PMC7191688

[32] Maraci M. A. Toward point-of-care ultrasound estimation of fetal gestational age from the trans-cerebellar diameter using CNN-based ultrasound image analysis. J. Med. Imaging 7, 1 (2020). 10.1117/1.JMI.7.1.014501 PMC6956669 31956665

