# Supplementary material for: Association between first trimester ambient temperature and the risk of gestational hypertension and pre-eclampsia: Insights from the PRECISE study cohort in The Gambia, Kenya and Mozambique
Source: Res Sq. 2026 Jul 29:rs.3.rs-10471213. Preprint. [Version 1] doi: 10.21203/rs.3.rs-10471213/v1 (PMC13484435; doi:10.21203/rs.3.rs-10471213/v1)
Supplement: Supplementary file 1 [file NIHPPRS10471213V1-supplement-1.pdf]

Supplementary Table 1: Table showing adjusted risk of gestational hypertension or pre-eclampsia per degree increase in the average maximum ambient temperature experienced in trimester one for the PRECISE pregnancy cohort [DAG covariates and full model with climatic zones] N indicated on each model

Multivariate analysis:	Measure of Association (95% CI)<br>aOR [c] (95% CI)	P-value<br>P-value
**Odds of** gestational hypertension or pre-eclampsia **per degree increase in average maximum ambient temperature in trimester one**<br>(Model **a**djusted for DAG variables:<br>Country, conception season (25th percentile vs 75th percentile temperatures), Gramin poverty index, N = 5489)	1.08 (1.04 - 1.12)	<0.001
**Odds of** gestational hypertension or pre-eclampsia **per degree increase average maximum ambient temperature Adjusted for DAG and a priori variables and climate zones:**<br>(Model adjusted for country, maternal age, Gramin poverty index, conception season (25th vs 75th), mother lives alone(yes/no), parity, maternal MUAC and HIV status<br>N = 5462	1.08 (1.04 - 1.12)	<0.001

Number (N) observations remaining in model, Measure of association **Odds Ratios** per model and respective 95% confidence intervals, p-values. Adjusted model covariates are indicated in each model description.

Supplementary Table 2: Table showing unadjusted and adjusted risk of gestational hypertension or pre-eclampsia per degree increase in the average maximum ambient temperature experienced in trimester one for the PRECISE pregnancy cohort stated for each exposure window, N indicated on each model

Exposure period	Measure of Association (95% CI)	P-value
**Preconception period**		
**Unadjusted analysis**	**OR (95%CI)**	**P-Value**
Odds of gestational hypertension or pre-eclampsia per degree increase in mean temperature in preconception (12 weeks before conception), N=6002	1.08 (1.06 - 1.11)	**<0.001**
**Adjusted analysis**	**aOR (95% CI)**	**P-value**
Odds of gestational hypertension or pre-eclampsia per degree increase in mean temperature in preconception adjusted for DAG and a priori variables: Country, maternal age, Gramin poverty index, (25th vs 75th), mother lives alone(yes/no), parity, maternal MUAC and HIV status [**excluding conception season**] N =5977	1.05 (1.01 - 1.08)	0.015
**Adjusted analysis**	**aOR (95% CI)**	**P-value**
Odds of gestational hypertension or pre-eclampsia per degree increase in mean temperature in preconception adjusted for DAG and a priori variables: Country, maternal age, Gramin poverty index, (25th vs 75th), mother lives alone(yes/no), parity, maternal MUAC and HIV status [**including conception season**] N =5476	1.02 (0.98 - 1.07)	0.372
**Trimester two**		
**Unadjusted analysis**	**OR (95% CI)**	**P-value**
Odds of gestational hypertension or pre-eclampsia per degree increase in mean temperature in trimester two, N=5992	1.06 (1.03 - 1.08)	**<0.001**
**Adjusted analysis**	**aOR (95% CI)**	**P-value**
Odds of gestational hypertension or pre-eclampsia per degree increase in mean temperature in trimester two adjusted for DAG and a priori variables: Country, maternal age, Gramin poverty index, conception season (25th vs 75th), mother lives alone (yes/no), parity, maternal MUAC and HIV status N = 5467	1.01(0.97 - 1.05)	0.677

Trimester three		
**Unadjusted analysis**	**OR (95% CI)**	**P-value**
Odds of gestational hypertension pr pre-eclampsia per degree increase in mean temperature in trimester three, N=5933	1.03 (1.01 - 1.06)	**0.006**
**Multivariate analysis:**	**aOR (95% CI)**	**P-value**
Odds of gestational hypertension pr pre-eclampsia per degree increase in mean temperature in trimester three adjusted for DAG and a priori variables: Country, maternal age, Gramin poverty index, conception season (25th vs 75th), mother lives alone (yes/no), parity, maternal MUAC and HIV status N = 5414	0.96 (0.93 1.0)	0.053

Number (N) observations remaining in model, Measure of association **Odds Ratios** per model and respective 95% confidence intervals, p-values. Adjusted model covariates are indicated in each model description.
